# What Recovery Means to Postpartum Women in Treatment for Opioid Use Disorder

**DOI:** 10.1089/whr.2021.0064

**Published:** 2022-01-31

**Authors:** Caroline Shadowen, Nancy Jallo, Anna Beth Parlier-Ahmad, Lisa Brown, Patricia Kinser, Dace Svikis, Caitlin E. Martin

**Affiliations:** ^1^ Virginia Commonwealth University Health System, Richmond, Virginia, USA.; ^2^ Department of Obstetrics and Gynecology, Virginia Commonwealth University, Richmond, Virginia, USA.; ^3^ Department of Family and Community Health Nursing, School of Nursing, Virginia Commonwealth University, Richmond, Virginia, USA.; ^4^ Department of Psychology, Virginia Commonwealth University, Richmond, Virginia, USA.; ^5^ Department of Family and Community Health Nursing, Institute for Women's Health, Virginia Commonwealth University, Richmond, Virginia, USA.; ^6^ Department of Family and Community Health Nursing, School of Medicine, Virginia Commonwealth University, Richmond, Virginia, USA.; ^7^ Institute for Drug and Alcohol Studies, Virginia Commonwealth University, Richmond, Virginia, USA.

**Keywords:** pregnancy, postpartum, opioid use disorder, qualitative, recovery

## Abstract

***Introduction:*** Opioid overdose has become a leading cause of pregnancy-associated deaths, particularly in the 1st year postpartum, highlighting the need to better understand how to promote recovery for postpartum women. This mixed-methods study aims to investigate how postpartum women receiving medication for opioid use disorder (MOUD) define recovery and factors associated with recovery progression or inhibition.

***Methods:*** Women receiving MOUD 2–6 months postpartum were recruited from an outpatient perinatal addiction clinic. Participants completed electronic measures including the Brief Assessment of Recovery Capital (BARC-10, total score range: 6–60) and semistructured individual interviews. Substance Abuse and Mental Health Services Administration (SAMHSA)'s recovery framework served as the conceptual model for interview guide development. Descriptive statistics were generated for survey responses. A qualitative descriptive approach was used to analyze and report the interview data.

***Results:*** On average, participants (*n* = 8) were 28.6 years old and taking 19.5 mg/day buprenorphine (range 8–24). Fifty percent identified as white and 37.5% as black. All participants identified as currently in recovery, with mean BARC-10 score 52.5 (standard deviation 4.8). Recovery goals included no use of drugs or alcohol (62.5%), being a better partner/spouse (87.5%), and improving finances (87.5%). Interviews generated themes including recovery as transformative, building resilience, and transforming one's health, relationships, and environment through recovery.

***Conclusions:*** Participants defined recovery as a dynamic transformative process, including nonabstinence-based goals consistent with SAMHSA domains coupled with reduced substance use. Central to recovery for our postpartum participants was the sense of self reinforced throughout their recovery journey. Women highlighted the key role of MOUD in their recovery process. Findings underscore the need for individualized treatment for postpartum women with opioid use disorder based on their personal goals and will inform development of a validated, gender-informed measure of patient-reported recovery outcomes tailored for this population.

## Introduction

In 2017, 2.8 million women met the criteria for substance use disorder (SUD).^1^ The intersection of women's unique health concerns and management of a chronic disorder make women's substance use a complex issue. For example, pregnancy is a time when women are supported with regular health care visits. Postnatally, women interface less with providers. These transitions, coupled with the increased stress and unique challenges of the postpartum period, make this point in the life course a vulnerable time. For women with opioid use disorder (OUD), transitioning from pregnancy to postpartum can be especially vulnerable.

Overdose has become a leading cause of pregnancy-associated mortality,^2^ with most deaths occurring after delivery, especially postpartum months 7–12.^3^ More research is needed to tailor approaches to optimize outcomes for women postpartum.^4^

In line with the Department of Health and Human Services' 5-Point Strategy to combat the opioid crisis,^5^ OUD treatment should prioritize recovery. Holistic recovery, including improved wellness and quality of life in addition to abstinence,^6,7^ is a complex concept influenced by self, situation, strategies, and support.^8^ Historically, we have understood recovery largely from the perspective of those who are not in specialized SUD treatment.

Recently, the Substance Abuse and Mental Health Services Administration (SAMHSA), a governing body for SUD treatment in the United States, developed a *clinically oriented* framework to enable care to better meet the needs of those receiving SUD treatment.^9^ SAMHSA defines recovery as “a process of change through which individuals improve their health and wellness, live a self-directed life, and strive to reach their full potential.” SAMHSA highlighted four domains of recovery: health, home, community, and purpose. SAMHSA's recovery framework can be useful to capture the multifactorial nature of treatment progress through postpartum.

The few studies of recovery among SUD treatment samples demonstrate that individuals experience recovery as multidimensional and transformational.^10,11^ Men and women experience recovery differently,^11^ with women typically having more social support, yet more robust trauma histories.^12^ The one qualitative study focused on postpartum found that pregnancy motivated women to seek recovery, and recovery transformed women's lives.^10^ Limited data exist regarding medical and social factors associated with recovery progression or inhibition postpartum, let alone how postpartum women define what recovery means to them. A critical step in prioritizing recovery in OUD treatment is to ask patients themselves how they define recovery.

The primary objective of this-mixed methods study was to investigate how postpartum women receiving medication for opioid use disorder (MOUD) define recovery. A secondary objective was to assess patient-reported promoting and challenging factors influencing recovery goal achievement.

## Materials and Methods

### Setting and design

This study was a component of a longitudinal study assessing treatment outcomes of postpartum women receiving MOUD at a perinatal addiction program. The program is affiliated with a large academic medical center in a Medicaid-expanded state, which serves as a safety net for the region and treats predominately individuals with low incomes, with many identifying as a racial or ethnic minority. Created in 2019, this perinatal addiction program is the only one in the region that provides an interdisciplinary integrated approach to women with SUD.

The program treats women across their life course, including during and after pregnancy and offers services such as treatment for SUD, medications such as buprenorphine/naloxone for OUD, obstetric care and recovery support during pregnancy, labor and delivery, behavioral health counseling, and referrals to other services.

The clinic prioritizes a low-threshold, harm reduction approach, meaning that established patients with recurrence of substance use are not exited from treatment but instead provided with increased wrap-around support and referral to a higher level of care. Patients in the program are at various stages of recovery and utilize different combinations of services available based on their needs.

For the parent study, women are recruited from this perinatal addiction program during the third trimester of pregnancy. A research assistant approaches each eligible patient in the clinic, describes the study, and invites the patient to participate. If a patient is interested and provides informed consent, she is enrolled and followed with monthly surveys through 12 months postpartum. Parent study inclusion criteria include the following: OUD diagnosis (The *Diagnostic and Statistical Manual of Mental Disorders*), receiving buprenorphine, 28–40 weeks gestation, age 18 years or older, and planning delivery at an affiliated academic medical center. Exclusion criteria include the following: severe psychosis, planning adoption, and non-English-speaking. Institutional Review Board approved all the study components. Participants provided verbal consent.

This study used a mixed-methods exploratory design to investigate recovery from the perspective of postpartum women receiving MOUD. Parent study participants were invited by a research assistant *via* telephone to participate in the current study once they reach 2–6 months postpartum. Of *n* = 12 eligible for participation, *n* = 8 participated.

### Data collection

Quantitative data were collected through a medical record review and an electronic survey completed before interviews. Participants reported their goals for recovery. Survey items also assessed recovery status using the Brief Assessment of Recovery Capital (BARC-10; total range 6–60, higher scores indicating more recovery capital)^13^ and Sheldon Cohen's 10-item Perceived Stress Scale (PSS; total range 0–40, higher scores indicate greater perceived stress).^14^

Qualitative data were collected by semistructured interviews; existing literature^15,16^ and research team experience supported the value of data from interviews within this patient population. The interview guide consisted of open-ended questions informed by SAMHSA's recovery conceptual framework.^1^ Interviewers asked prompts to define recovery and used probes as needed to ensure input on each recovery dimension (full interview guide in Supplementary Table S1).

All participants completed interviews by Zoom. Two or three moderators conducted the interviews: C.S. (medical student with clinical addiction experience) and N.J. and/or L.B. (researchers with qualitative experience). Interviews were audio-recorded and lasted 30–60 minutes. One moderator took notes during interviews.

### Data management and analysis

Descriptive statistics were generated for demographic, clinical, and psychosocial characteristics as well as self-reported recovery status and goals. Group mean BARC-10 and PSS scores are reported.

We determined our sample size based upon Lincoln and Guba's guidelines regarding “informational redundancy,” through which when no new information was obtained, then interviews could be concluded.^17^ In addition, we take seriously the concept of “information power,” through which when powerful data are obtained from each individual participant, then a small sample size may be adequate. As such, based on our team's extensive experience in qualitative research, we determined that after eight participants completed interviews, a point of informational redundancy and information power had been reached.^18^

Our small sample of participants provided rich, powerful data.^19,20^ Specifically, our sample provided sufficient information power for the aims of the study according to Malterud et al.'s five criteria.^21^ First, our study aim was narrow, as we aimed to investigate recovery using a multidimensional framework, yet only from the perspective of postpartum women receiving a specific SUD treatment: MOUD.

Second, the combination of study participants was appropriately specific for the study aim; all participants were women with OUD who continued buprenorphine from the pregnancy through the postpartum period and rated their recovery high. Simultaneously, our women also exhibited sufficient variation in their experiences through their recovery trajectories, achieving an excellent balance in sample specificity.

Third, our study procedures from conception through analysis were guided by an established theoretical framework: SAMHSA's recovery conceptual framework. This allowed us to synthesize our interview questions and findings efficiently.

Fourth, interview dialogue was strong between researchers and participants which translated into high analytic value of our data. Lastly, our intended analysis strategy was to elucidate themes of recovery *via* in-depth exploration of our participants' stories rather than a broader cross-case analysis; our small sample size was well suited for this analysis plan.

The interview data were analyzed using a qualitative content analysis approach.^22–24^ This approach allowed for participants' data to provide a comprehensive description of their individual experiences, facilitating a greater understanding of what recovery means to postpartum women in the treatment for OUD. This approach was determined to be suitable for the study, as the intention of the study was to obtain postpartum women's definition of recovery, thereby focusing on aspects of meaning, experience, and understanding.^25^ The qualitative content analysis method^26^ also facilitated the determination of themes related to SAMSHA's recovery framework in the participants' experiences.

Directed content analysis allows for a structured deductive approach to qualitative data. It involves coding, data reduction, and identification of themes in relation to predetermined content categories.^27^ Although based on the content or concept, this type of concept analysis allows for induction of new categories relevant to the framework, which makes it useful in extending knowledge about a particular phenomenon. This approach permitted analysis of specific aspects of the SAMHSA's recovery framework that are of interest to the research team, as well as recognition of new patterns in the data.

The analysis process consisted of several steps: once digital audiofiles were transcribed into Microsoft Word, (1) authors C.S., N.J., A.B.P.A., L.B., and C.E.M. individually reviewed each transcript line by line; (2) the same authors reread the transcripts line by line and assigned codes to key concepts that arose in the data; (3) this team met to discuss topics that emerged from the data and using an inductive process created a category theme list to organize the data; (4) these authors individually rereviewed transcripts to ensure the themes accurately represented the data and then finalized themes and identified quotes to highlight the themes.

Thematic analysis allowed themes and subthemes to emerge independently of the SAMHSA domains, although the authors also noted where our findings overlapped with SAMHSA categories. Authors P.K. and D.S. served as peer debriefers, reviewing the analysis themes and processes, to ensure confirmability of the data and enhance rigor of the study.^28^

## Results

### Quantitative

Mean age of the sample was 28.6 (standard deviation [SD] 1.7) years. Mean daily dose of buprenorphine was 19.5 (SD 5.7, range 8–24) mg. On average, women had 2.0 (SD 1.9) living children (Table 1).

**Table 1.** Sociodemographic and Clinical Characteristics of Participants (*N* = 8)

**Participant characteristics**	***n* (SD)**
Months postpartum at study completion	3.5 (1.7)
Mean age ± SD (years)	28.6 (5.5)
Race/ethnicity	
White	4
Black	3
More than one race	1
Insurance status	
Public	7
Private	1
Pregnancy history	
Total number of gestations	3.1 (2.0)
Total number of living children	2 (1.9)
History of C-section	2
History of preterm delivery	3
Medical/psychiatric history	
Any medical diagnosis	6
Any psychiatric diagnosis	8
Substance use history	
History of injection drug use	1
Amount of time on MOUD at study completion (months)	16.1 (10.2)
Buprenorphine dose at most recent clinic visit (mg/day)	19.5 (5.7)

MOUD, medication for opioid use disorder; SD, standard deviation.

**FIG. 1.**
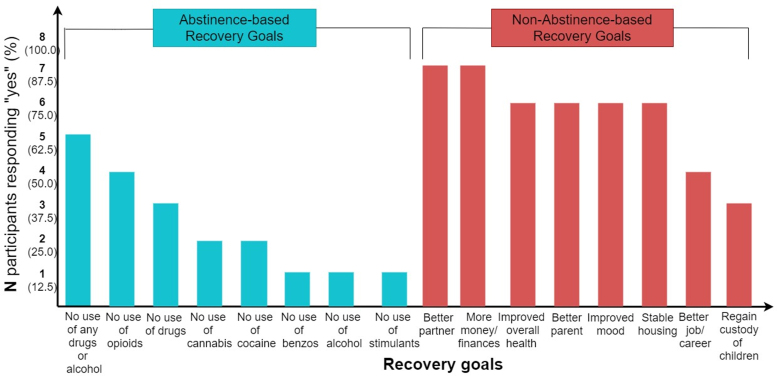
Graph of abstinence- and nonabstinence-based recovery goals reported in quantitative surveys. Teal bars represent abstinence-based goals; red bars represent nonabstinence-based goals. *y*-Axis indicates number (*n*) and percentage (%) of respondents who responded “yes” to each goal. *x*-Axis lists recovery goals in descending order of number of respondents answering “yes.”

All participants felt they were currently in recovery, with mean BARC-10 score 52.5 (SD 4.8) and PSS 14.4 (SD 6.6). Participants reported abstinence- and nonabstinence-based recovery goals (Fig. 1). Five of eight indicated a long-term recovery goal of “no use of any drugs or alcohol.” Nonabstinence-based goals included becoming better partners (7/8) and more money/better finances (7/8). Seven participants reported being treated rudely or disrespectfully at some point due to substance use.

### Qualitative

**FIG. 2.**
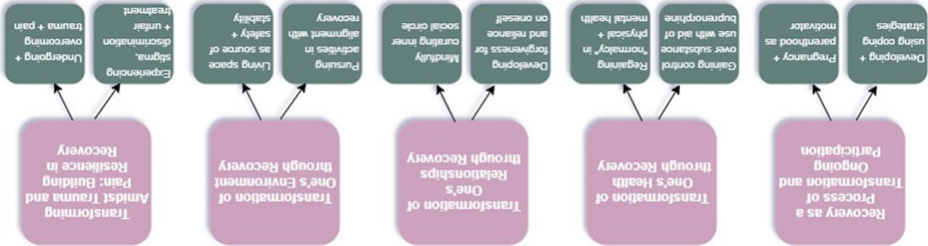
Figure summarizing main interview themes and subthemes from qualitative results. Main themes are represented in pink boxes, with arrows indicating subthemes in green boxes.

Five themes emerged from interviews: (1) recovery as a dynamic process of transformation requiring ongoing participation; (2) transformation of one's relationships through recovery; (3) transformation of one's environment through recovery; (4) transformation of one's health through recovery; and (5) building resilience amidst trauma and pain. Figure 2 summarizes qualitative results; Supplementary Table S2 lists additional representative quotations.

### Recovery as a process of transformation and ongoing participation

A prevailing theme was the transformational nature of recovery across all aspects of life: “recovery is when you have completely flipped your life around from what it was” [A]. Participants defined recovery as “a way of life” [B] that required them to “change your whole way of thinking” [B].

Pregnancy was highlighted as a catalyst for recovery. Participants stated, “when I got pregnant with my first, my life completely changed…That was the biggest thing that started this jump [recovery]” [C] and “my son absolutely saved my life” [H]. Women developing identities as mothers/parents motivated them during and after pregnancy: “putting in perspective that you're a mother and that [your kids] need you, it's actually changed recovery to ‘I never want to touch anything again. I want to understand myself. I want to love myself’” [C]. Motherhood provided purpose in recovery: “my purpose, I try to be the best mother I can…if I was still using, I would not be the mother I am right now” [D].

Participants recognized the fragile nature of recovery, commenting, “it's a struggle every day” [E] and “in the blink of an eye, it could be taken from you. It is not promised. It can be gone within a minute” [G]. Interviewees emphasized how they made choices through a recovery lens: “I just don't have time for anything that's not really about my recovery” [G]. This theme of constant vigilance was common: “I gotta pay attention to [my recovery] and…not ignore it and think I'm alright. Because I could not be okay” [A].

Because of the fragile nature of this process, women readily recognized their accomplishments, stating, “I'm doing great” [C], “I am doing this every day” [E], and “[recovery] is just something that makes me stronger” [E]. One participant expanded on this sense of achievement: “this is the best my life has been in probably 10–12 years…I just had the baby, I just bought myself a car. Before, I could not do that…My life is the way a normal life should be, and it's amazing. I'm very happy” [A].

Participants actively engaged in their ongoing recovery transformation by learning and utilizing wellness tools. One participant noted, “in the group I was in, they called [coping strategies] mooring lines, to think of [yourself] as a boat…to stop and check these things and make sure everything is where it should be to keep you on the right path” [H].

Women engaged in strategies such as identifying substance use triggers, setting boundaries, and self-care. They identified “stretching and yoga and massage” as helpful for physical symptoms of anxiety, and “counseling” [A], “group therapy” [H], and “positive affirmations” as helpful for mental symptoms. Rather than using substances to “mask the emotions” [D] of anxiety and depression, participants learned to “deal with real life” [E].

A few participants described recovery more literally, as “staying clean, refraining from using any drugs” [D] and “getting and staying clean and eventually getting off Suboxone” [H].

### Transformation of one's health through recovery

Recovery transformed participants' mental, physical, and spiritual health. They had “to be ready to replace everything…If you don't have the faith in your mental, in your health, you're not going to do it. For me, my mental helped me a lot, and my health” [G]. These positive health changes motivated women: “health has been the main thing that has kept me going…I'm a strong female…What motivates me is my health” [G]. Participants mentioned how “not having cravings or being able to effectively control them and not having withdrawal symptoms” [H] provided a foundation in recovery to “effectively handle stressors moving forward” [H].

MOUD was a major component of recovery. Participants noted how “this medication has changed my life” [A] and that “as long as I am taking that, I'm in my right frame of mind, and I'm able to think clearly as to why I should not use [substances]” [C]. MOUD enabled participants to succeed in recovery: “my family loves Suboxone. They see such a positive change in my life and the way I am every day” [D]. A few women mentioned how “a lot of people talk about the Suboxone. They say, ‘you're just substituting one drug for another’” [A] but were quick to point out that, despite experiencing stigma from others, they take MOUD “by prescription, through a doctor. And it saved my life” [A].

Transformation of physical health was common. One participant noted, “I've been eating. In active addiction, I would not eat…[Now] I eat regularly and I look healthy and I have energy. I go to bed at a decent time. I'm not up all hours of the night…I'm not hurting. I'm not sick. It's amazing being able to wake up in the morning and not be sick” [A].

Reciprocally, recovery and mental health transformed: “before recovery, my stress and anxiety were out of this world, but now that I'm in recovery…my anxiety is much better because my whole life did a 360 turn-around” [F]. In addition, “putting the major focus on my mental health” [H] was cited as “a big reason why I've been successful with this whole process” [H]. Several participants described how “recovery has enhanced my feelings” [E], forcing them to face emotions they became numb to during active addiction.

### Transformation of one's relationships through recovery

Women in recovery mindfully curated their social network and noted the transformational influence of recovery on relationships. Sometimes, the relationships that initially triggered substance use became sources of motivation and strength in recovery: “my mom is a recovering addict. Seeing [her] go through the stuff she went through and then seeing how she quit was really my motivator, even though she was one of the reasons why I started doing drugs because of all the things I went through as a child” [G]. Prioritizing recovery required participants to “love [some old friends] from a distance” [H] when they realized “friends I once had are still doing things they should not do and I don't want to be a part of” [C].

A strong relationship with oneself was central to continued success in recovery. One participant stated, “I can't talk about my support system without including myself because I've really had to do a lot of work and self-improvement and soul searching on myself in the process” [H]. Recovery and self-love were reciprocally related: “[My recovery] just made me a very strong person. When you're using, you tend to feel very hopeless and worthless. As you continue in your recovery, you find how to love yourself” [C].

Although all participants acknowledged external motivators, they emphasized that ultimately, recovery required recentering and reprioritizing themselves: “Recovery is for yourself. That's just a bonus point that you can be a better mother to your kids. You just got to get yourself together first, or you're not gonna be able to be there for the kids” [C]. This self-acceptance included facing mistakes they made during active addiction: “the hurt that I've caused to others…gives me more motivation to try to prove that I'm doing what I'm supposed to do” [A] and “in recovery, you have to face all these mistakes you made and you gotta try to slowly get things back and make amends with people” [B].

### Transformation of one's environment through recovery

Participants sought safety and security in their homes and neighborhoods, underscored by the need to “stay away from the opiates and out of the environment and people and stuff that put me there” [F]. Several women began their recovery journey in settings that isolated them from substance use, such as recovery houses where they “couldn't leave, couldn't go anywhere because of how the program was set up” [C] or “a human trafficking shelter [where] I stayed all the time. That helped me not give in to cravings because I was in this place. I was only allowed to talk to my family, so I didn't have an opportunity to use [substances] even if I wanted to” [H].

Consistently, participants changed their environments to maintain recovery: “where I was living before was really rough…[Now] my house has been a big sanctuary for me. I just lock myself away…I don't have any temptations around me, no problems, nothing” [G], and “for my dad to let me live in my grandma's home is a big deal because I wasn't allowed to come anywhere near this house when I was using” [A].

Participants highlighted the impact of environments outside their homes. For example, one woman chose not to return to a previous job, noting, “I've learned that in recovery, until I feel at a stable point, I will not go find employment. Work was one of my triggers. I was a waitress, so being around alcohol was the trigger…It seems riskier to me early on in my recovery to get a job where I could be stressed out to the point of relapsing” [C].

Employment did align with some women's recovery. One participant found “purpose at work…I love my career, I love my job” [A]. Another works “doing hair full-time. I feel much better doing it. I'm exceeding what I planned to do…I want to one day open up my own salon” [F]. Participants engaged in activities aligned with recovery and/or disengaged from activities that did not.

### Transforming amidst trauma and pain: building resilience in recovery

For most women, traumatic experiences and pain impacted addiction and recovery courses. One mother who survived sexual violence noted, “even though I was already using, when things like that happen, it makes you want to use more and more until it just goes away, until you don't think about that stuff” [E]. A mother who survived human trafficking described telling herself, “this happened to me, so I deserve to get high a couple of times” [H]. Another who experienced multiple miscarriages noted that going through something painful “either emotionally or physically” was a “big trigger” for her, and she described her miscarriages as “my relapse” [A].

Women highlighted the importance of overcoming traumatic experiences as part of recovery. Participants described “tools in the toolbox” [H] to help them cope. One mother who survived intimate partner violence explained, “once I get overwhelmed with that type of stuff, I do tend to think about using again. But…having that self-control and reminding myself that I have a lot to lose if I relapse, I continue to keep that in mind, as well as my children” [D]. Women overwhelmingly demonstrated resilience in recovery.

All participants experienced discrimination due to substance use. Women described family members, friends, and community members who were “very, very rude, and mean and nasty” [A] about their addiction, contributing to feelings of shame and helplessness. One woman explained, “people turn their nose up because they knew I was an addict…It makes you feel terrible about yourself. That could make you give up on trying to get help. You feel worthless” [C].

Women in recovery noted the “strong head” [G] and perseverance required to overcome internalized stigma. One woman stated, “what other people think about me doesn't matter because if my husband's happy with me, family is happy with me, and I'm doing a good job with the kids and the house…I just have to keep patting myself on the back and not needing that from other people” [B]. Others noted that their recovery progress “shows itself, with what I have and who I am now. It kind of shows the success of the hard work I have put in” [A].

Women experienced stigma related to MOUD, recounting comments such as “you're not clean because you're on Suboxone” [A] and receiving MOUD is “switching out one addiction with another.” Participants described judgment from health care providers and denial of appropriate medical treatment due to SUD, especially for medical problems with associated pain (*e.g.*, kidney stones). They recalled being perceived as “just here for drugs” [F], or being told they were careless or “abusive” [H] mothers. In these situations, participants advocated for themselves and their children and focused on moving forward. One participant noted, “It's hard to not be heard or be taken seriously because of your past and the mistakes you made. But you can't let it affect you. You can't let that win. You have to just say, ‘that's who I was. That's not who I am’” [B].

## Discussion

This mixed-methods study investigated how postpartum women receiving MOUD define recovery. We integrated concurrent quantitative and qualitative data to describe what recovery means to this unique sample, using in-depth interviews to expand upon and better understand self-reported survey data.

**FIG. 3.**
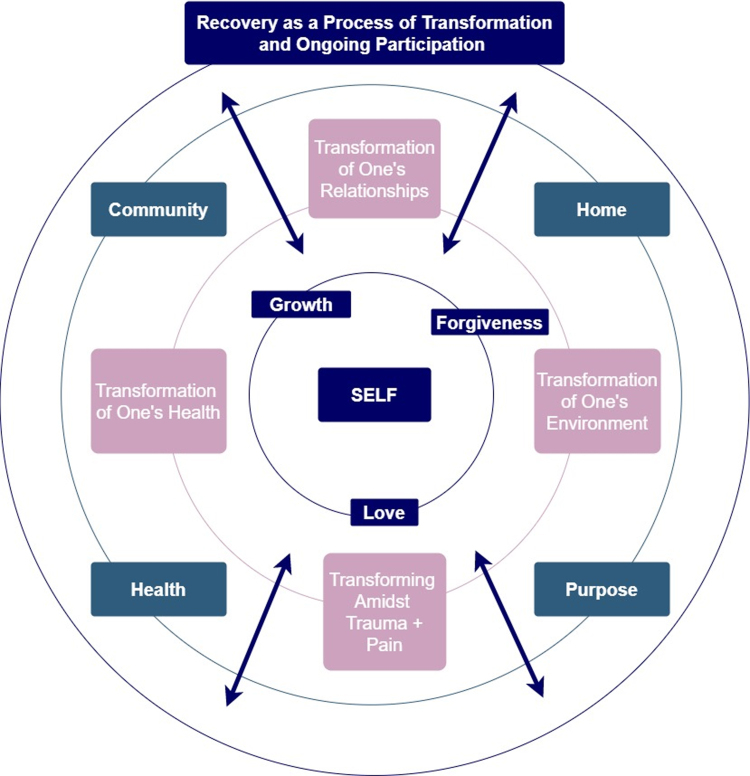
Figure representing findings from our mixed-methods data overlaid with SAMHSA recovery domains. Inner circle with indigo boxes represents themes and subthemes from current study findings. Inner middle circle with pink themes from current study findings. Outer middle circle with teal boxes containing SAMHSA domains. Outer circle with indigo box representing current study finding theme. Arrows within figure demonstrate bidirectionality between all components of recovery. SAMHSA, Substance Abuse and Mental Health Services Administration.

Our sample of women receiving MOUD and successfully maintaining recovery postpartum defined recovery as a dynamic process of transformation across all areas of life. They highlighted aspects of recovery from the postpartum perspective, underscoring supportive relationships, engagement in meaningful activities, physical and mental wellness, and resilience. Participants reported nonabstinence-based recovery goals coupled with reduced substance use, consistent with SAMHSA's recovery domains (Fig. 3). Accordingly, we advocate for individualizing treatment for postpartum women with OUD based on their definition of recovery and unique strengths and challenges.

Consistent with SAMHSA's framework, our participants' health improved in recovery and enabled recovery progress. MOUD maintenance was critical to enhancing health and reducing substance use.^29^ Previous qualitative research indicates that people in recovery frequently manage comorbid physical and mental health diagnoses, and women often have a higher incidence of self-harm and suicide attempts than men.^11^ Our participants disclosed experiencing traumas, which are more prevalent in women (*e.g.*, sexual assault and miscarriage).^30–32^

Findings provide additional support for trauma- and gender-informed recovery treatment, especially late in the 1st year postpartum when depression, anxiety, and suicidality increase.^33^ Ongoing communication with peer support, therapists, and physicians throughout the postpartum period may support new mothers.^34^ Such multidisciplinary care should extend beyond a typical 6 weeks of follow-up after delivery to adequately support women through this vulnerable transition.^35^

Similar to previous studies, our participants noted the importance of safe and stable home environments. Neighborhood safety can impact outcomes for women in recovery,^36^ and establishing a routine within the home may aid recovery journeys.^12^ The positive effects of stable living environments extend beyond women in recovery, improving outcomes for their child(ren)'s development and overall well-being of their families.^37,38^ Evaluating postpartum women for housing stability and providing resources to those with housing instability may mitigate challenges for some women.

Finding purpose in recovery is a dynamic process. Participants described how pregnancy was an initial motivator for recovery, and how their developing identities postpartum as mothers have strengthened their recovery. Pregnancy is a window of opportunity to engage women with OUD in treatment,^39^ and existing research demonstrates that becoming mothers can be transformative for many women in recovery.

Previous qualitative studies of women with SUD^40–42^ note that becoming pregnant and/or having children serve as a catalyst for recovery, transforming personal identities to recovering parents.^42^ Of course, purpose described by our participants and in the literature extends beyond motherhood. Meaningful roles such as serving as peer support for others in recovery can help sustain personal recovery.^8^ Finding purpose within or outside the home impacts recovery success; postpartum women should be given the opportunity to reflect on what their individual purpose may be.

Our participants frequently mentioned their communities: friends, families, partners, doctors, therapists, fellow mothers in recovery, and other supporters. The breadth and depth of support may be an advantage, as one study demonstrated that individuals in recovery benefit from a range of relationships, both “closer to” (*e.g.*, partner) and “further from” (*e.g.*, coworkers) the individual.^8^

Our participants unanimously mentioned needing to stop contact with friends and family members who were not healthy for their recovery. This can be especially difficult for women who have a limited social circle and can vary according to cultural norms and expectations.^40^ Being a woman may be protective in the social domain of recovery as women may have an easier time forming new recovery-oriented friendships than men and experience less social isolation.^11^ SUD treatment providers may serve as support to new mothers in recovery as they make necessary changes to their social circle and offer opportunities to develop new healthy relationships (*e.g.*, moms' group).

For our sample of postpartum women, central to all SAMHSA domains was the role of “self.” Through identifying and developing individual strengths, admitting to and forgiving themselves for past shortcomings, and acknowledging the strength required to overcome trauma, all participants developed a relationship with themselves as a fundamental component of their recovery. Women had motivation and support from outside forces and acknowledged that they alone were responsible for choosing to enter and continue to prioritize recovery.

Similarly, prior qualitative research found that postpartum women with SUD and depression felt more confident and successful when they entered treatment for themselves rather than involuntarily.^43^ Women are aware of how delicate recovery is and express a sense of accomplishment from creating success for themselves.^12^ Further research is needed to evaluate the roles of love, forgiveness, and growth of self in recovery, and to develop a clinically useful way to evaluate sense of self in recovery.

There are limitations to our study. We only included women currently in SUD treatment receiving MOUD at a single institution in North America, limiting generalizability. Only participants who enrolled and remained in the larger longitudinal study through at least 2–6 months postpartum were eligible for the current study, inserting selection bias into results.

Our selective sample is also a strength, as our findings are pertinent to patients in integrated obstetrics and gynecology-Addiction programs, increasingly more prevalent in the overdose crisis. A strength of our study is that our participants all reported being in recovery and had high levels of recovery capital, demonstrating that postpartum women receiving MOUD can successfully sustain recovery. Our findings shed light on these women's success, allowing us to learn from their experience to expand the evidence base to improve treatment for all postpartum women with OUD.

## Conclusions

This mixed-methods study demonstrated that, for our sample of postpartum women receiving MOUD, recovery is an ongoing process of transformation extending beyond reduced substance use and consistent with SAMHSA's four recovery domains: health, home, community, and purpose. Central to recovery for our postpartum sample is the foundation of “self,” which women developed by identifying individual strengths, overcoming trauma, accepting their pasts, and developing self-reliance and self-love. Of note, all participants highlighted the key role of MOUD in recovery. Future research should focus on developing and evaluating recovery outcome measures and tailored interventions specific to postpartum women with OUD.

## Supplementary Material

Supplemental data

Supplemental data

## Abbreviations Used

BARC-10: Brief Assessment of Recovery Capital
MOUD: medication for opioid use disorder
OUD: opioid use disorder
SAMHSA: Substance Abuse and Mental Health Services Administration
SD: standard deviation
SUD: substance use disorder
