# Supplementary material for: What Recovery Means to Postpartum Women in Treatment for Opioid Use Disorder
Source: Womens Health Rep (New Rochelle). 2022 Jan 31;3(1):93–103. doi: 10.1089/whr.2021.0064 (PMC8812494; doi:10.1089/whr.2021.0064)
Supplement: Supplemental data [file Suppl_TableS1.docx]

*Supplement 1*. Full interview guide for qualitative component, consisting of general questions about recovery, questions in each of the SAMHSA domains, as well as probing questions within each domain.

**Recovery**

| How do you define recovery? |
| --- |
| What are the most important components of recovery to you? |
| How do you feel like you’re doing in your recovery? Why is that? |

**Health**

| How does your health, including physical, mental, emotional, and spiritual aspects, affect your recovery? How do these things help your recovery? How do they limit your recovery? |
| --- |

*Medication*

| What role, if any, has medication played in your recovery? |
| --- |

*Pain*

| How do you feel pain affects your recovery? |
| --- |

*Mental Health*

| How does your stress, anxiety, depression, or other mental health disorder affect your recovery? How does your recovery affect your mental health? |
| --- |

**Home**

| How does your home environment affect your recovery? |
| --- |

**Purpose**

| How would you describe your sense of purpose in your recovery? |
| --- |
| How has your recovery affected your sense of purpose? |

*Parenthood*

| How has pregnancy/motherhood impacted your definition of recovery? |
| --- |
| How do you feel your identity as a parent or a mother affects your recovery? |
| How has your recovery affected your identity as a parent? |

*Work*

| What role, if any, does employment play in your recovery? |
| --- |

**Community**

| How do you feel your larger community of neighbors, coworkers, fellow people at Motivate affects your recovery? |
| --- |
| How does your recovery affect your ability to engage with your community? |

*Relationships*

| Who is/are source(s) of support in your recovery? |
| --- |
| Who is/are source(s) of challenge to your recovery? |
| How has your recovery affected your relationships? |

**Other**

| How have you experienced stigma or discrimination? |
| --- |
| How has stigma or discrimination played a role in your substance use or recovery? |
