# Supplementary material for: What Recovery Means to Postpartum Women in Treatment for Opioid Use Disorder
Source: Womens Health Rep (New Rochelle). 2022 Jan 31;3(1):93–103. doi: 10.1089/whr.2021.0064 (PMC8812494; doi:10.1089/whr.2021.0064)
Supplement: Supplemental data [file Suppl_TableS2.docx]

*Supplement 2.* Additional representative quotations from participant interviews by domain.

| ***Recovery as a Process of Transformation and Ongoing Participation*** |
| --- |
| [Recovery] is hard work…it’s because of me…nobody’s done it for me. |
| [Recovery is] a life changer. |
| You have to change your whole thought process, along with everything else in recovery, because taking responsibility for your actions is really hard to do. |
| There’s just so much more to recovery than just not using. |
| It's just a whole thought process change, trying to stay positive, not feeling sorry for yourself. Not blaming everybody all the time for your mistakes. It’s called ‘growing up.’ |
| Having all the tools that you need to fight cravings and handle stressors and things like that, different ways to calm yourself down and deal with just the different stresses of everyday life without wanting to run back to using. |
| I go to sleep when I'm really upset with my boyfriend. Or I'll take a shower. Showers help. |
| I feel like [parenthood] gives me more reason to stay clean because I've got not only myself to take care of, I’ve got a lot of other people relying on me now that I'm back in their life… I want to stay clean so I can be there for them. |
| If I'm not clean, then I really can't do nothing for myself and I can’t do for my kids. I can't be the best mother I can be for my kids. I can't be the best person that I know I can be for myself. |
| My children are my purpose in my life, and their father. I strive every day for them. |
| My purpose in life is being a mother. I absolutely love it. I was told I wouldn’t be able to have kids, and then she came into my life, and it was like a miracle. I turned my whole life around for her. |

| ***Transformation of One's Health through Recovery*** |
| --- |
| My health actually is better, to be honest because before when I would do the opiates and when I couldn't get them, I would be sick. It wasn't a great feeling at all. I would be sick, couldn’t do anything at the time. So I'm much better now. |
| Suboxone has played a huge role in me and my husband's life. If I hadn’t had that….It reduces cravings for not just opioids. So I have no desire or even think about it. |
| I would recommend [Suboxone] honestly, because I've tried everything over the last 10, 12 years and nothing has worked. So it's a big, big difference. |
| Being in recovery, I’ve had to focus on making long term solutions instead of short, self-medicating ways to get rid of those things. It helps completely in the long run. Also, obviously, being on Suboxone and being in recovery and not having to worry about where I'm going to get my next fix or where I'm going to get the money to get my next fix or finding somebody to get it from or worrying about when withdrawal symptoms are going to start – that takes a major stress off of you mentally. Anyways, I have more energy, I feel better. I have more patience, I’m not so quickly angered as I used to be at one point. I feel like just in general, a combination of everything that I've been doing has really had a positive impact on my life. |
| I'm doing really well. I walk around the block with my daughter every evening and take her somewhere every day to get her out of the house. I sleep well, I eat well. I'm very grateful. |
| I’m a little bit more overweight than I would like to be. But my husband says, “leave it alone.” He hasn't ever wanted me to be ninety-seven pounds again. He's in recovery also. But everything health is good. |
| I’ve been seeing a therapist once a week. And that helped because anything that had been on my mind, even if it was just one time and it really, really bothered me, I was able to talk that out with her versus sitting on that thought and it continuously bothering me. |
| I feel like I can't think straight sometimes because I'm just so overwhelmed with that feeling of stress or anxiety. And that's when I'm like, “alright, let me go ahead and catch it now before it gets way out of hand.” Like I said, it's taken me about ten years to get to this point, though. It's not been easy, but I can gather all of that from the past and use that experience for now, and I think that's why I've been successful. |

| ***Transformation of One's Relationships through Recovery*** |
| --- |
| Being in recovery has positively affected how I communicate with others. |
| Working on trust in those relationships, I've definitely seen a huge improvement. But I have a lot of work to go and I’m willing to do that because these relationships are important to me. |
| [My recovery] just made me a very strong person. When you're using, you tend to feel very hopeless and worthless. But as you continue in your recovery, you find how to love yourself. |
| I can't talk about my support system without including myself, because I've really had to do a lot of work and self-improvement and soul searching on myself in the process. |
| A lot of mothers tend to think they're the only ones going through certain situations, they’re the only ones that did the horrible things that come with drug use. But being in groups of other mothers, you find out that everyone's done basically the same things. |
| I wasn't forced into recovery, they never gave me an ultimatum. It's just unconditional love. I probably wouldn't be alive if it wasn't for my grandmother constantly helping me. |
| My fiancé…I can talk to him about anything. He supports everything that I do. He will do whatever he needs to to help me, which is awesome. He's been with me for four years now. He was with me when I was using, and then he's with me when I'm clean. So he stuck around. I'm very, very thankful. |
| These people I've been friends with for all these years, it's not disloyal to them that I don't want to be around them. It's actually loyal to myself. |
| My family is not the best for my recovery. Just being that I only have my mom and my sister. They both use…You can't really look to them as role models or anything like that. |

| ***Transformation of One's Environment through Recovery*** |
| --- |
| Before I was in recovery, I would literally stay home alone because I was living by myself, all by myself. I would stay to myself, wouldn’t talk to anybody, wouldn’t do anything, wouldn’t get out of bed. Now, I get up and I get out of the house every day or I’ll go to my daughter or work. So being in recovery has positively affected my whole life. |
| I do my [dog] grooming thing…I come in on time. I never call out. Before, when I was using, I would have called out. I would have been 30 minutes late every day. I would have taken long lunches. |
| But when [my son] gets a little older, I plan on working to keep myself occupied, busy. My mind won’t have enough time to think about other stuff besides my children, my home, my man, and work. And I plan on going back to school. |
| I think that this environment (living with mom and stepdad) for the beginning of my recovery is probably the best for me because I'm not alone. Because I've done that in the past, having my own place and so on and so forth. And I can get away with murder if I wanted to, I don't have anybody kind of keeping me in check…I have plenty of that here. |
| As far as the neighborhood, I'm not around the people that I used to be around. No drama, nobody trying to bring me down, nobody trying to offer me anything. I don't have to worry about waking up and seeing a bunch of addicts running around. I don't have the temptation. |
| With any home, you have to make sure it's in the right area so that you don't feel pressure because you're in that area. You also want to think of, "are there people that are going to trigger me to get high in that area?" If you're in an unsafe home or anything, that's obviously not the best choice. |
| Inside my home is wonderful, but where I live, I really don't love it. It's a lot. There's a lot of things that go on where I live at and currently I'm trying to find another place, so I can really, really flourish. |
| In early recovery, some of the worst things that you have to deal with are like changing the people around you in your life and the situations and the places you go because anything can be a trigger. |

| ***Transforming Amidst Trauma and Pain: Building Resilience in Recovery*** |
| --- |
| When I was younger and using, I always looked to my mom for the reassurance of the person I was dating or I always let her pick the guys for me to date. It was terrible. She always chose the ones that were either selling drugs or getting high…when I got sober and clean, I saw what she was doing and it made me sick. I felt like I was being sold…When it comes to guys, that's the only thing I've ever had a problem with, was guys wanting to put their hands on me, especially when I was using…then being told that, if you didn’t want it to happen, then why would you dress like that or why’d you do your hair like that or your makeup?...a lot of guys, they looked at me like a toy or a whore, and it was really just a bad time in my life…Even though I was already using, when things like that happen, it just makes you want to use more and more until it just goes away, until you don't think about that stuff. It's hurtful. It takes a lot to get over, a lot of drugs to get over. But no matter how many drugs you use, pain is always there. |
| In the beginning, especially getting out of the situation that I was in, it's easy to use those kinds of things…Like, this happened to me, so I deserve to get high a couple of times. It's easy to use those things as excuses. But by doing counseling and really focusing on my mental health helped me a lot with the recovery process because a lot of those triggers and wanting to use is really based on other things that are going on. So, focusing on reigning those in and getting all that stuff under control has helped me a lot. |
| One of the worst things that could happen to anybody was losing someone you love. Someone I love died right next to me. And that's what scared me into getting clean, wanting to go down the right path. I don't really know how to define recovery. In my case, I was scared. I was scared into recovery in a sense. |
| I've been discriminated against even in my recovery. One of my close friends…told me, “well, even though you're clean, you're going to always just be an addict anyway. You can always just relapse at any time.”…I think my life kind of shows itself, with what I have and who I am now. It kind of shows the success of that, the hard work that I have put in. So, if somebody starts to talk crap, I'm like, “well, you know what? I don't care, because I know at the end of the day, I'm doing what I'm supposed to do.” |
| There's that stigma that people don't recover, that everyone's gonna relapse, a junkie is a junkie. But that's very untrue. We may relapse, we may fall short sometimes, but we're always going to pick ourselves up. |
| I did feel like that it was immediately assumed that my child was withdrawing and that I wasn’t taking seriously what I'm putting in my child's body because I'm just an addict, and I don’t think that way. “People like us” just don't think…It was very hurtful, and it really upset me…It was very sad to see him suffer longer than he needed to get the proper treatment before people would finally let go… It is hard to not be heard or be taken seriously because of your past and the mistakes you made. But you just can't let it affect you. You can't let that win. You have to just say, “that's who I was. That's not who I am, and nobody can convince me otherwise.” |
| Yeah, when I was pregnant with [my son] and I was giving birth, right after I had him, I was in so much pain. I didn't know why I was in so much pain. I kept telling the doctors, “I'm in pain.” They were looking at my file, and I was upfront and honest with them that I was in recovery and that I was an opiate addict. But when I told them that I was in pain, they were just basically making me feel like I was trying to get pain medication. And then they sent me home in all that pain. When I went to go see my OB almost a month later, I had so many built up of hematoma, like blood clots. It was so out of hand and I didn't understand why they just didn't believe me when I told them I was hurting but it was because in my file it said I was an opiate addict. It was really unfair. |
| I was having problems healthwise. I actually had to have surgery last year for it, but I was literally having problems with my uterus and my ovaries…I would literally be in pain and when I went to the hospital, they would be like, “oh, you’ve been red-flagged, we can't give you anything for pain because we feel like you’re just here for pain medicine, blah blah blah.” …One lady at the hospital said loudly in front of everyone, “You’re just here for drugs.” What? I’m literally hurting, literally had to have surgery… It really hurt my feelings, but I think that I refused to let it get to me. |
| They were like, “you don't look like a heroin addict.” What does a heroin addict look like? What does that look like? What does addiction look like? It can look like anything. There's no set way somebody should look. I don't have needle marks all over my arms because that’s not how I did it. There’s so many different ways somebody in active addiction could look… It's very easy for people who have never been in somebody's shoes to judge their journey and how they move forward. |
